# Infrared Irradiation of H_2_O:CO_2_ Ice: A Combined Experimental and Computational Study of the Dissipation of CO_2_ Vibrational Excitations

**DOI:** 10.1021/acsearthspacechem.5c00030

**Published:** 2025-05-23

**Authors:** Johanna G. M. Schrauwen, Tobias M. Dijkhuis, Sergio Ioppolo, Daria R. Galimberti, Britta Redlich, Herma M. Cuppen

**Affiliations:** † 6029 HFML-FELIX Laboratory, IMM, Radboud University, Toernooiveld 7, 6525 ED Nijmegen, The Netherlands; ‡ Institute of Molecules and Materials (IMM), Radboud University, 6525 ED Nijmegen, The Netherlands; ¶ Leiden Institute of Chemistry , Gorlaeus Laboratories, Leiden University, 2300 RA Leiden, The Netherlands; § Leiden Observatory, Leiden University, 2300 RA Leiden, The Netherlands; ∥ Centre for Interstellar Catalysis (InterCat), Department of Physics and Astronomy, University of Aarhus, Aarhus DK-8000, Denmark

**Keywords:** astrochemistry, ices, infrared irradiation, free electron laser, experiments and simulations, classical molecular dynamics, vibrational energy dissipation, mixed ice

## Abstract

In interstellar ices, the ice matrix can have a great influence on the chemical reactions. The hydrogen-bonding network in pure water ices facilitates fast energy dissipation that, for example, stabilizes the HOCO complex, a crucial step in the formation of CO_2_. To better understand the energy dynamics and its possible influence on the processes in the ice, we investigated a H_2_O:CO_2_ 1:4 ice mixture exposed to infrared irradiation on-resonance with the CO_2_ vibrations. Experimentally, we find changes in the OH stretch of H_2_O after irradiating the asymmetric stretch of CO_2_ for several minutes with the intense monochromatic light of the FELIX free electron lasers. Using molecular dynamics simulations, we found that an excitation of the asymmetric stretch of CO_2_ readily dissipates to other asymmetric stretches in the environment, but only dissipates to the CO_2_ libration and H_2_O twist modes after roughly 2 ns because of its minimal anharmonicity and coupling with other modes. This is significantly longer than the off-time between laser pulses of 1 ns, suggesting ladder climbing or that the stacking of the excitation boosts the experimentally observed changes. For infrared excitation of the CO_2_ bending vibration, the simulations reveal a fast distribution of energy and coupling to the intermolecular interactions that lead to thermal heating of the H_2_O vibrational modes. This is not observed on the time scale of the experiments. Still, both simulations and experiments reveal nonthermal annealing of the H_2_O component of the mixed ice when exposed to infrared irradiation on-resonance with the CO_2_ vibrations.

## Graphical abstract

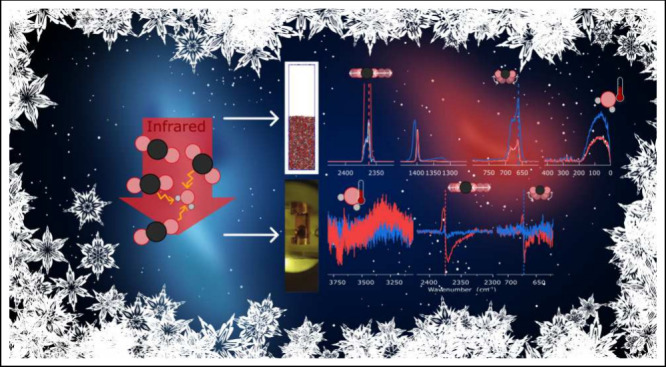

## 1 Introduction

Interstellar ices play an important role in the solid-state chemistry of the interstellar medium and are crucial to advancing the chemical complexity in space. These ices – any solid in space that would be a gas or liquid on Earth, such as CO_2_ and H_2_O – are formed at low temperatures on the surface of interstellar dust grains through a series of different ‘(non)­energetic’ processes. In the early stages of molecular cloud development, it is assumed that the first ice to form in this translucent environment is made of H_2_O molecules.^1^ The gas phase at this point is still mainly atomic. In this stage, oxygen and hydrogen atoms freeze-out onto interstellar grains and react to form H_2_O molecules, together with smaller amounts of carbon and nitrogen atoms that can form CH_4_ and NH_3_ upon hydrogenation.^2–4^ Such atom-addition surface reactions involve energies of the order of a few meV and therefore are considered ‘nonenergetic’ compared to reactions induced by cosmic rays, ultraviolet photons, and electrons with energies from a few eV up to GeV. When the density in the cloud increases, all the heavier species in the gas phase freeze-out, and at this stage, CO molecules form a coating layer on water-rich ice grains. The result is an often observed two-layer ice system consisting of a polar phase (mainly H_2_O) and an apolar phase (mainly CO).^5–7^

Depending on the cloud region and the phase of the star-forming process, these ice layers can be exposed to nonenergetic atom bombardment or energetic processing, such as cosmic ray and UV irradiation and thermal heating.^8^ Together with the accumulation of chemical species, this can drive rich chemistry and lead to the formation of so-called complex organic molecules, such as glycine, which may be precursors to biologically relevant species.^9–11^ An important early step in this process is the formation of CO_2_, one of the most abundant molecules in space and the main subject of this study.^12–15^

The formation of CO_2_ in the gas phase is relatively inefficient and, therefore, it is expected to form in the solid state.^16^ CO_2_ is detected in both the polar (water rich) and apolar layers (CO rich) of the interstellar ices,^17^ which is well-connected to the observation that it should be formed from a component associated with CO (CO or HCO) and one of H_2_O (O or OH). Experimental and theoretical work showed that the nonenergetically driven reaction to CO_2_ proceeds through the HOCO complex.

This HOCO complex is efficiently stabilized in a H_2_O environment, preventing subsequent dissociation to CO_2_ directly.^18^ This stabilization can be attributed to the efficient energy dissipation dynamics previously found in liquid water, crystalline water ice, and in porous amorphous solid water (pASW).^19–21^ In the latter case, molecular modeling revealed that the hydrogen-bonding network is the primary carrier of fast energy dissipation after excitation with an electric field on resonance with the OH stretch: an energy range similar to the excess energy of many reactions. The hydrogen bonds themselves do not get excited, but they rapidly transfer the energy of the excitation to neighboring molecules with similar vibrational frequencies. Any defect sites in the modeled pASW hampered the energy dissipation.

Energy dissipation also plays an important role in the competition with chemical desorption – ejection of a freshly formed product into the gas phase caused by the excess energy of a reaction – that is considered essential to explain the gas-phase abundance of interstellar complex organic molecules that are believed to be formed on the icy grain. For example, during the formation of NH_3_ on a water ice surface through successive H atom addition, quantum mechanical simulations found that 58%–90% of all energy released during the reaction is absorbed within 1 ps by the ice surface, resulting in a temporary increase of the ice surface temperature, but not desorption.^22^ The energy dissipation occurred through a coupling of the vibrational modes of the newly formed species with the water libration modes, as well as a coupling of the NH, NH_2_ and NH_3_ bending modes with the H_2_O bending modes. For the hydrogen-atom addition to CO a similar effect was observed, where the crystalline ice surface absorbed 90% of the energy in the first picosecond, leaving the HCO-radical product with too little energy to desorb.^23^ Molecular dynamics simulations have also shown that for H_2_O, CO_2_ and CH_4_ desorption can only occur through translational excitations, whereas chemical reactions generally supply vibrational excitation.^24^ Dissipation of vibrational energy to the surface occurs readily, but no interconversion between translation, vibrational and rotational energy was found. Most of these studies consider pure H_2_O as the ice surface, inspired by the H_2_O-ice abundances in the interstellar medium, and the hydrogen-bonding network appears to play an important role in the fast energy dissipation.

Inspired by the role of the ice matrix structure in the formation of HOCO and the crucial role of energy dissipation in various interstellar reactions, we began to explore how specific defects in the hydrogen-bonding network, created by the incorporation of other molecular species, influence these processes. While many previous studies have focused on a pure water surface, where the hydrogen-bonding network is vital for efficient dissipation, in this paper we study the effect of a weakened network. In our previous experiments using infrared irradiation from a free electron laser (FEL), which is a suitable probe for energy dissipation dynamics in pASW,^21,25^ we found that although the hydrogen-bonding network was weakened with the inclusion of CO_2_ in the water ice, the dissipation mechanism remained the same upon excitation of the OH stretch of H_2_O.^26^ For these mixtures, we did not have comparable simulations, like for pASW in the work from Cuppen et al.^21^ As a result, we were unable to access the molecular level of the process, which restricted the analysis to macroscopic observations. And as our analysis had to rely on previous pure pASW studies, we could not extensively analyze the H_2_O-poor mixtures.

In this paper, we investigate a H_2_O-poor mixture of CO_2_ and H_2_O on which we performed both FEL irradiation experiments and simulations. Specifically, we study an amorphous H_2_O:CO_2_ mixture with a mixing ratio of 1:4 to sufficiently dilute the hydrogen bonding network, but to maintain an amount of H_2_O in the mixture that can still be observed in the infrared along the strong and narrow CO_2_ vibrations. We study a range of thicknesses of the amorphous mixture to investigate surface and bulk effects.

## 2 Experimental and Computational Methods

### 2.1 Experiments

Mixed H_2_O:CO_2_ 1:4 ices of different thicknesses are deposited in the Laboratory Ice Surface Astrophysics (LISA) ultrahigh vacuum chamber with a base pressure of 1 · 10^–9^ mbar at room temperature, stationed at the HFML-FELIX laboratory in Nijmegen, The Netherlands. A detailed description of the LISA chamber as sketched in Figure 1 will be published elsewhere, and here we restrict our discussion of the experimental procedure to the characteristics of the current experiments.

**Figure 1:**
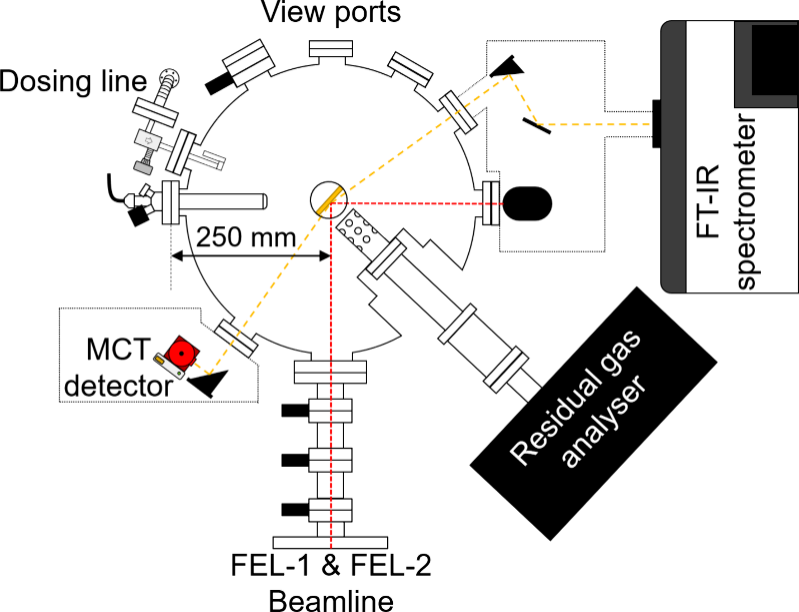
Schematic top view of the LISA chamber stationed at the HFML-FELIX laboratory in Nijmegen, The Netherlands.

The gold-coated copper substrate is cooled to the lowest achievable temperature of the cryostat, about 9 K for the current configuration, to produce amorphous ices. Prior to deposition, H_2_O (deionized and purified via multiple freeze–pump–thaw cycles) and CO_2_ (≥99.995%, CANgas, Sigma-Aldrich) gases are mixed in the stainless steel dosing line. The mixing ratio of 1:4 H_2_O:CO_2_ is controlled using two mass-independent gauges with overlapping ranges of 0.001–10 and 0.1–1000 mbar. Four different ice thicknesses are chosen; 27 L, 53 L, 107 L, and 360 L, with 1 L corresponding to 1 s of background (nondirect) deposition at a pressure in the chamber of 1 · 10^–6^ Torr with 1 Torr corresponding to 1.3 mbar. During all depositions, performed at a pressure of 1 · 10^–6^ mbar, the growth of the ice is monitored by Fourier-transform reflection absorption infrared (FT-RAIR) spectroscopy recording a sequence of spectra every 15 s in the 5000–500 cm^–1^ range with a resolution of 0.5 cm^–1^ and 8 coadded scans.

After deposition, the ices are allowed to stabilize for approximately 10 min, while the background gas is pumped away and no differences are observed in RAIR spectra taken 5 min apart. After the stabilization period, the ice is irradiated with the intense, monochromatic, and tunable infrared light of the FELIX free electron laser (FEL) 2. FEL-2 is a pulsed laser that, for these experiments, produces macropulses of ∼6 μs at 10 Hz that are carried by micropulses fired at 1 GHz. Irradiations are performed for 2.5 min on-resonance with the CO_2_ asymmetric stretch at 4.215 μm and bend at 14.88 μm with an average macropulse energy of 67 mJ and 91 mJ, respectively. The full width at half-maximum (FWHM) of FEL-2 is less than 0.025 at 4.215 μm and about 0.070 at 14.88 μm. The FEL-2 beam irradiates the sample at an angle of 45°, irradiating an elliptical spot of about 0.2 mm^2^ at 4.215 μm and about 1.7 mm^2^ at 14.88 μm. All irradiations are performed at the same temperature as the depositions and all data was obtained within one FELIX beamshift.

Due to experimental and time constraints, the substrate cannot be cleaned of the ices of the previous thickness between irradiation experiments. Therefore, we use a vertical-translation stage allowing for 11 irradiations at nonoverlapping positions on the ice. The thinnest ice is deposited on the clean substrate, and after irradiation with 4.215 μm at one of the 11 positions, a new deposition is performed on top of the first ice while the substrate is back in its central position. The resulting thicker ice layer is then irradiated at 4.215 μm on a new spot on the substrate. This process, depicted in Figure 2, is repeated until all four thicknesses are studied. After completion of the experiments on the four thicknesses, the substrate is cleaned by heating the substrate until the ice has fully desorbed (180 K), and the series in Figure 2 is repeated for irradiations at 14.88 μm. To ensure that the layers all have the same mixing ratio, they are deposited from the same gas-phase mixture.

**Figure 2:**
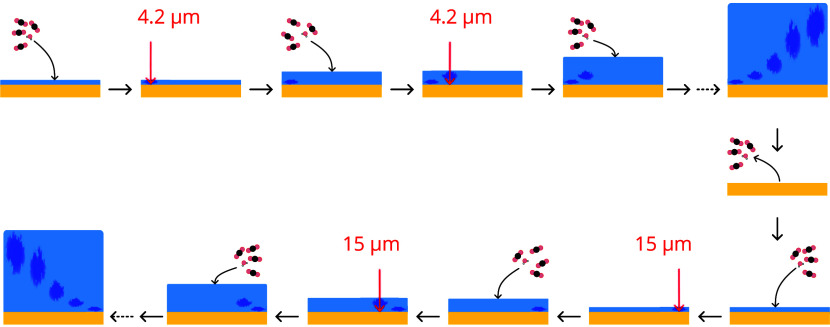
Illustration of the experimental procedure to perform irradiations with different frequencies (from two different FEL energy ranges) and ice thicknesses within one FELIX shift (8 h). The ice is deposited in steps, building a thicker layer with every step. Between depositions, irradiations are performed at a specific height on the substrate to preserve enough ‘pristine’ ice for the next deposition and irradiation. For the 15 μm irradiation, the FEL had to operate in a different energy range, and, as such, these irradiations are performed in a second sequence after clearing the substrate.

To study the effect of infrared irradiation, FT-RAIR spectra are taken just before and just after the irradiation, in the range of 5000–500 cm^–1^ with a resolution of 0.5 cm^–1^ and a total of 256 coadded scans. Subtracting the two spectra will result in a difference spectrum, highlighting the changes in the shapes of the vibrational bands. During irradiation, the gas phase is monitored by a residual gas analyzer (Hiden HAL3F PIC) operating in multiple ion detection (MID) mode, tracking *m*/*z* 18 and 44 as a function of time with a dwell and settle time of 5 ms. Measurement of these masses at ∼50 Hz allows for at least 5 data points within the time interval between macropulses.

### 2.2 Simulations

To model the irradiation of the H_2_O:CO_2_ 1:4 ices, classical molecular dynamics (MD) simulations are performed using the LAMMPS Molecular Dynamics Simulator, version 28 March 2023.^27^ For CO_2_, intermolecular and intramolecular parameters are taken from Zhu and Robinson^28^ and the COMPASS force field,^29^ respectively. The two models rely on different atomic charges, and those of the EPM model of Harris and Yung are adopted for these simulations. The intramolecular potential does not only contain harmonic terms for the bond stretch, but also third and fourth power terms, as well as cross terms. The intermolecular and intramolecular interactions in H_2_O are modeled using TIP4P/2005f, the flexible version of TIP4P/2005, required to adequately model the vibrations of H_2_O.^30^ The pair potential of CO_2_–H_2_O is taken from Karssemeijer et al.^31^ The accuracy of the resulting force field is checked by comparison with the experimental spectra of the mixtures.

The amorphous mixed ice model is created in two steps, starting from a cubic box with a length of 1200 Å filled randomly with a total of 4000 CO_2_ molecules with a minimum spacing of 2 Å with periodic boundary conditions. After an initial minimization, 1000 H_2_O molecules are added in random positions with the same 2 Å spacing. To decrease the distance between the molecules, the box size is first halved and, after another minimization, the box size is reduced to 85.5 Å in 20 ps. During this process, tip4p/long interactions are disabled to prevent *k*-point selection issues due to the rapid change in box dimensions. Stable ice at 10 K is obtained by cooling to 100 K in 10 ps, followed by a quench to 20 K in the *NPT* ensemble until the cell lengths are constant (within 2500 ps), and results in cell lengths of *a* = *b* = *c* = 59.7 Å, which corresponds to a density of 1.52 g cm^–3^.

The infrared FEL irradiation is simulated with an oscillating electric field of an on-resonance frequency with the simulated CO_2_ asymmetric stretch and bending mode at 2355.16 cm^–1^ and 656.94 cm^–1^, respectively. The electric field is applied on the majority of the cell, except for a small spherical cluster of 50 molecules. The electric field is maintained for 4 ps with an intensity of 0.04 V/Å on the CO_2_ asymmetric stretching vibration and 0.02 V/Å on the CO_2_ bending vibration. The system is then allowed to relax for a preset time after which the velocities of the 150 atoms within the cluster are recorded every 1.5 fs for 200 ps to calculate the mass-weighted velocity autocorrelation function to obtain the vibrational density of states (VDOS). All simulations are performed within the microcanonical ensemble (*NVE*) and a 0.5 fs time step. The simulations are visualized with VMD^32^ and analyzed using in-house Python scripts.

## 3 Results

### 3.1 Characteristics of the Experimental Ice Analogues

Before discussing the effect of the irradiations on the experimental ice analogues and the simulations, we discuss the characteristics of ices deposited in the LISA chamber. Figure 3 shows the RAIR spectra of the eight investigated interstellar ice analogues. Since each thickness is deposited twice to allow for irradiation on the two different vibrational modes of CO_2_, Figure 3 shows two similar traces per thickness (light-colored trace: CO_2_ asymmetric stretching irradiation, dark-colored trace: CO_2_ bending irradiation). The ices are deposited such that two depositions of the same thickness are spectrally as similar as possible. The CO_2_ asymmetric stretch is very sensitive to its environment, so slight differences in the position of the stretch peak are observed between depositions of the same thickness. For the two thicker ices, 107 and 360 L, the peak shift is about 0.5 cm^–1^, which is the resolution of the spectrometer. The shift is closer to 1.5 cm^–1^ for the thinner ices. These shifts are likely caused by a gradual enrichment of the background gas with CO_2_ and mainly H_2_O, since the depositions are performed sequentially. This effect is minimized by employing the maximum possible waiting time between subsequent depositions, but this cannot recreate the conditions of the first deposition performed 3 days after the last deposition in the chamber. Therefore, the shift is the largest for the thinnest ice that is deposited first in both deposition series. Still, compared to the FEL’s FWHM of 30 cm^–1^ in this range, this shift is negligible and should not significantly influence the result of the irradiation. Additionally, the first depositions of the 80 and 360 L ices suggest a slightly more intense OH stretch that is probably related to the slightly longer deposition times for these ices, as shown in Table 1.

**Figure 3:**
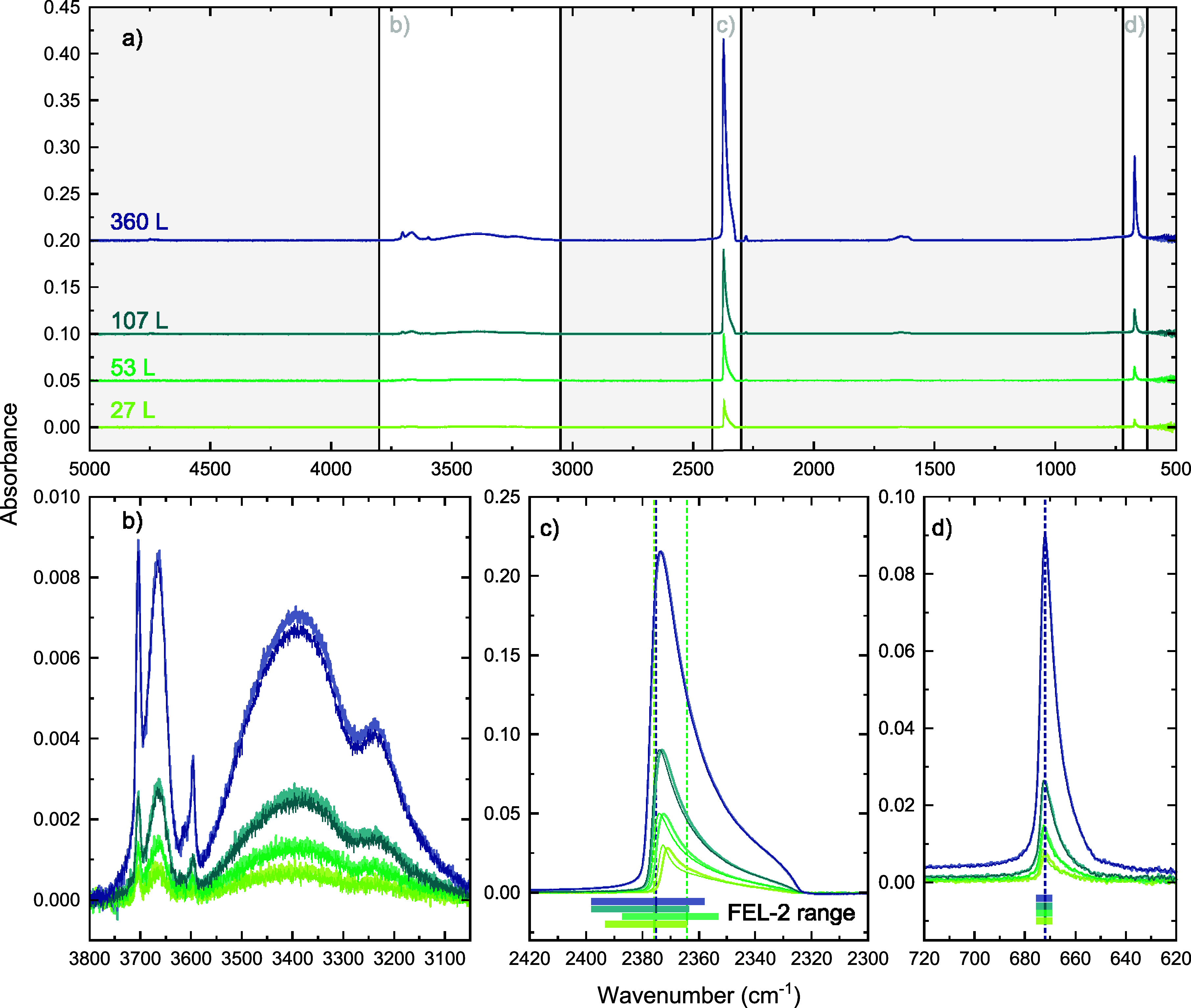
Infrared spectra of the four ices with different thickness after deposition (27 L, 53 L, 107 L, and 360 L). Panel a) shows the full spectral range and b), c), and d) zoom in on the relevant vibrational modes, i.e., the OH stretch of H_2_O, the CO_2_ asymmetric stretch, and the CO_2_ bend, respectively. In panel b) we also observe the first CO_2_ combination, dangling OH of H_2_O and second CO_2_ combination modes from left to right to the left of the broad OH stretch of H_2_O. Each thickness was deposited twice to allow for irradiations at the CO_2_ asymmetric stretch (light-colored trace) and the CO_2_ bend (dark-colored trace). Panels c) and d) include the total spectral width of the FEL-2 beam in the horizontal bars for irradiation at the CO_2_ asymmetric stretch and the CO_2_ bend. The vertical dashed lines indicate the central frequency of the FEL. The spectra of the different thicknesses are vertically offset for clarity.

**Table 1:** Calculated Mixing Ratios and Thicknesses of the Eight Deposited Ices

Deposition (L)		H_2_O:CO_2_ ratio		Thickness (nm)	
Aim	Actual	Gas-phase^b^	Solid-state^c^	2.7L = 3.5 Å	Band strength
27^a^	27.5	4.44	4.11	3.61	0.88
	28.1	4.49	4.57	3.68	0.86
53	54.8	4.38	4.17	7.19	1.69
	55.3	4.43	4.50	7.26	1.66
107	109.0	4.36	4.20	14.3	3.20
	108.7	4.41	4.50	14.3	3.14
360	370.8	4.90	4.37	48.8	8.71
	367.9	5.02	4.59	48.3	8.64

a The first row per aimed deposition corresponds to the ice deposited for irradiation at the CO_2_ asymmetric stretching vibration and the second row to the ice deposited for irradiation at the CO_2_ bending vibration.

b Mixing ratio in the gas phase during deposition.

c Mixing ratio in the ice.

To obtain an estimate of the actual thicknesses and mixing ratios of the eight ice samples, we calculate both the mixing ratio and thickness in two ways. The thickness we can estimate from the deposition time and from the band strength of the water OH stretching and CO_2_ asymmetric stretching vibrations taken from transmission experiments, but corrected for RAIR spectroscopy using the procedure described in Ioppolo et al.^33^ The mixing ratio of the ices is determined from the gas-phase ratio as recorded by the residual gas analyzer during deposition and the ratio of the column densities calculated from the band strength of the H_2_O and CO_2_ asymmetric stretching vibrations. The results are listed in Table 1.

For the calculation of the thickness of the ice from the band strength measured in transmission experiments, we determine the column densities from the OH stretch of H_2_O with $A_{3297{cm}^{-1}}=1.5\cdot 10^{-16}$ cm/molecules and the CO_2_ asymmetric stretch of CO_2_ with $A_{2343{cm}^{-1}}=7.6\cdot 10^{-17}$ cm/molecules.^34^ To convert column densities to thicknesses, we use a density of 0.87 g cm^–3^ for amorphous H_2_O and 1.11 g cm^–3^ for amorphous CO_2_.^34^ The correction of the column densities for our RAIR spectroscopy configuration with an angle with the substrate surface of 10^*o*^ is sin­(θ)/2 = 0.087. Since RAIR and transmission spectroscopy are not directly comparable, thicknesses calculated in this way should be considered estimates, and better RAIR correction factors should be obtained in future studies from laser interference techniques that are currently not available on the LISA setup. In addition, the band strength of CO_2_ is uncertain, and multiple different values are reported in the literature.^34–36^ Moreover, the transmission band strength and densities used are for pure ices, yet here we use them to describe a mixture.

To estimate the thickness of the ice in another way, we perform repeated temperature-programmed desorption (TPD) measurements, as shown in the Supplementary. Based on the transition from first-order to zeroth-order desorption kinetics, we conclude that for the LISA setup a single monolayer of CO_2_ corresponded to a deposition of about 2.7 L. From the density of our simulated ice, we determine that a monolayer should correspond to 3.5 Å. Hence, we can estimate the thickness of the ice using 2.7 L = 1 ML = 3.5 Å. This monolayer thickness obtained from the simulations is close to the estimated monolayer thickness from a simple back-of-the-envelope calculation adding the experimental densities of the pure ices, which is 3.8 Å.

Table 1 shows the thickness and mixing ratio estimates for the eight ices. The solid-state ratios are close to the desired 1:4 ratio, but the thickness of the ice is unclear. There is roughly a factor of 4 between the thickness estimated from the number of deposited monolayers and the thickness calculated from the band strength and density in the literature. According to the solid-state spectra, the ices are much thinner than the thicknesses expected from the deposition time. This is likely due to the many assumptions involved in calculating the solid-state thickness from the infrared spectra, especially regarding the RAIR spectroscopy correction factor. However, since we restrict our discussion of the thickness dependence in this paper to qualitative observations, the variety is more important than the actual thickness.

### 3.2 Experimental Infrared Irradiation

The deposited ices are irradiated on-resonance at the CO_2_ asymmetric stretching and bending vibration, and the frequency overlap of the Gaussian FEL-2 beam is shown in the colored horizontal bars in Figures 3c) and d). For the irradiation at 4.21 μm the FEL-2 beam is slightly asymmetrical with respect to the highest intensity of the beam and the spectral width and frequency changed slightly during the experiments. Such small variations and slight asymmetry is more common for experiments around 4.21 μm. The results of these irradiation experiments are shown in Figure 4 as difference spectra – the difference between a RAIR spectrum recorded before and after irradiation. The top panel of Figure 4 shows the full difference spectra, and the bottom panels show details of the changes in the specific vibrational modes. Some narrow peaks are observed at 4425, 2870, and 1890 cm^–1^ with similar intensity in almost all difference spectra. These are noise peaks resulting from vibrations in the spectrometer. The difference spectra in Figure 4a) labeled with double stars show decaying desorption peaks of *m*/*z* 44 (CO_2_) in the MID recorded during irradiation. The difference spectra labeled with single stars only show a faint hint of desorption; see Supporting Information.

**Figure 4:**
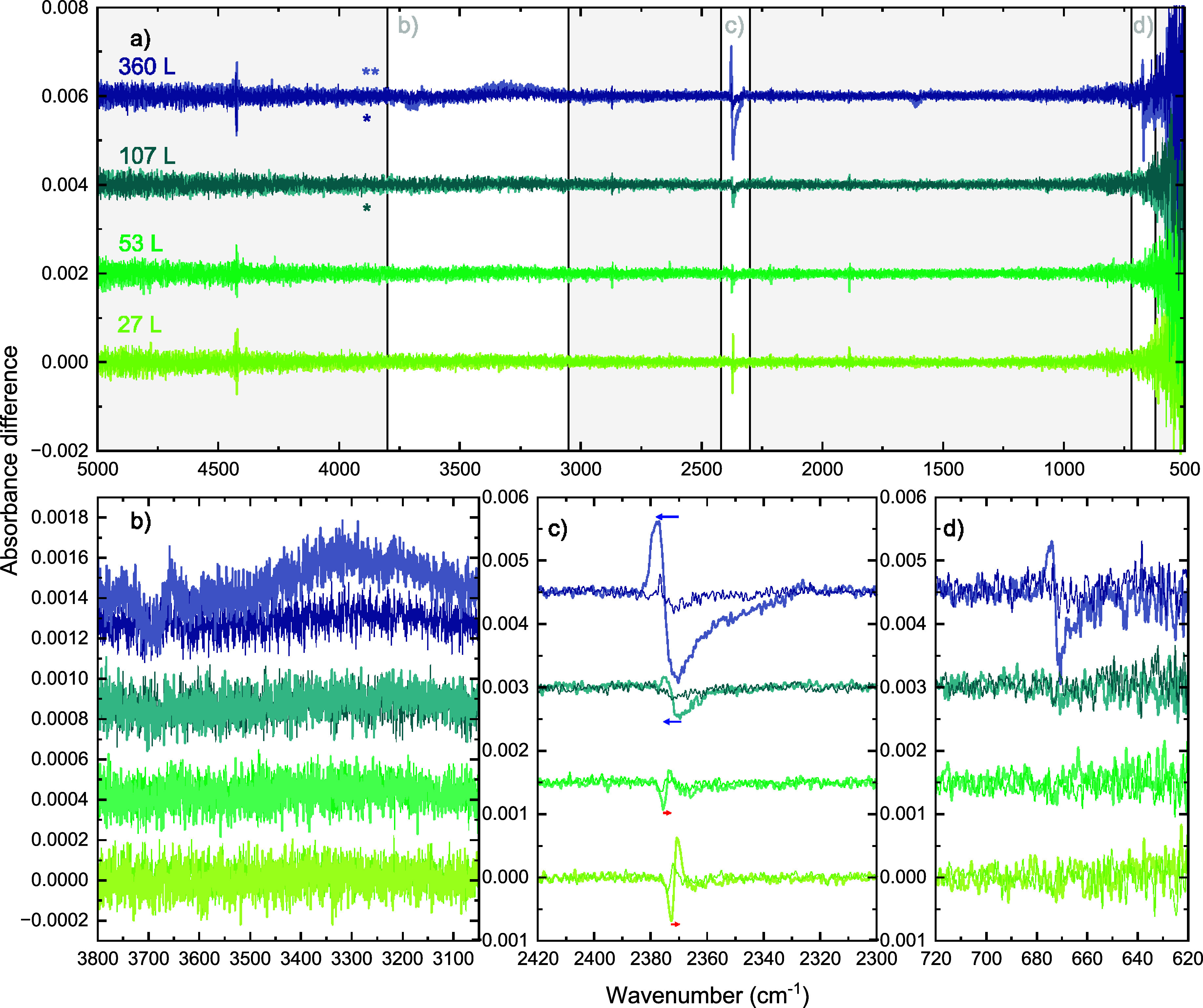
Infrared difference spectra showing the structural change after irradiation for the four ices with different thicknesses (27 L, 53 L, 107 L, and 360 L). Panel a) shows the full difference spectra, and b), c), and d) zoom in on the changes in the relevant vibrational modes, being the OH stretch of H_2_O, the CO_2_ asymmetric stretch, and the CO_2_ bend, respectively. Irradiations of 2.5 min at 10 Hz were performed at the CO_2_ asymmetric stretch (thick, light-colored trace) and the CO_2_ bend (thin, dark-colored trace) with the FEL spectral width and overlap as indicated in Figure 3. The double stars in panel a) indicate irradiations that resulted in multiple desorption spikes, and those labeled with a single star resulted in a few desorption spikes. The red and blue arrows in panel c) highlight the red-shift and blue-shift profiles in the restructuring of the CO_2_ asymmetric stretch. The difference spectra of the ices of different thicknesses are offset vertically for clarity.

Irradiation at the CO_2_ bend (dark-colored traces in Figure 4) does not result in significant observable changes in the difference spectra. No changes are observed in the OH stretch of H_2_O, nor in the CO_2_ bending mode. Weak changes resembling the irradiation of the CO_2_ asymmetric stretch (light-colored traces in Figure 4) can be observed in the CO_2_ asymmetric stretch. These have, however, a too low signal-to-noise to be adequately analyzed.

Irradiation at the CO_2_ asymmetric stretch (light-colored traces in Figure 4) results in clear changes in the CO_2_ asymmetric stretch itself. For all thicknesses, the change in the CO_2_ asymmetric stretch consists of both a positive and a negative part, indicating that the ice restructures. The areas of the negative and positive parts vary between thicknesses, with the strongest changes visible in the irradiation of the thickest ice of 360 L. Interestingly, the weakest changes are not observed in the thinnest ice but in the 53 L ice. We will investigate changes in the CO_2_ asymmetric stretch in more detail in the next section. Changes in the bending vibration upon irradiation of the stretch are visible only for the thickest ice. Changes can also be present for the other thicknesses, but the higher noise of our infrared detector in this region limits their observation.

Most strikingly, irradiation at the asymmetric stretch of CO_2_ appears to induce changes in the OH stretch of H_2_O for ice of 360 L thick. The difference spectrum shows an increase of intensity in the OH stretch, indicating an increase in bulk-H_2_O interactions in the ice. In addition to the increase, the OH-dangling region shows a narrow negative signal, indicating the loss of surface modes. This can potentially result from segregation, leading to the clustering of H_2_O molecules in the CO_2_ environment. Segregation can occur naturally after the CO_2_ molecules in the vicinity desorb as detected by the mass spectrometer, allowing the now neighboring H_2_O molecules to interact. However, restructuring could also indicate an energy dissipation mechanism between the vibrational modes of CO_2_ and H_2_O in the amorphous ice mixture.

This restructuring in the OH stretch is only observed for the thickest ice, which can be related to the mechanism that induces the change, but it is more likely that the change is not visible for thinner layers because of the experimental signal-to-noise ratio. For thin ices, Figure 3b) shows that the OH stretch is not very intense and, especially for the 27 L one, almost within the noise level. A small change in the structure of such a weak band will not be visible above the signal-to-noise ratio, even though it is present. This could be the case for the thin ices, but in the current setup, we do not have the means to improve this.

To better understand the microscopic changes that carry the observed spectral change in the OH stretch, we use a Gaussian oscillator fitting code.^25^ Each Gaussian oscillator describes a specific hydrogen-bonding environment, and by fitting the difference spectra, we can relate the spectral change to the local hydrogen-bonding environment of the H_2_O molecules, as described for pure porous amorphous solid water by Noble et al.^25^ The heights of the fitted Gaussians are shown as a function of the hydrogen bonding environment in Figure 5 and represent the change in population in the specific environment. Here, we denote accepting hydrogen bonds with A and donating hydrogen bonds with D, such that DAA refers to a water molecule with three hydrogen bonds of which one is donating and two are accepting. The fit in Figure 5 shows that the increase in the OH stretch can be attributed to a loss of doubly hydrogen-bonded (DA) and triply hydrogen-bonded (DAA and DDA) species and a gain in quadruply hydrogen-bonded molecules (DDAA). We investigate how the energy input in the CO_2_-vibrational modes results in this restructuring in the OH stretch on the molecular level with the simulations in Section 3.4.

**Figure 5:**
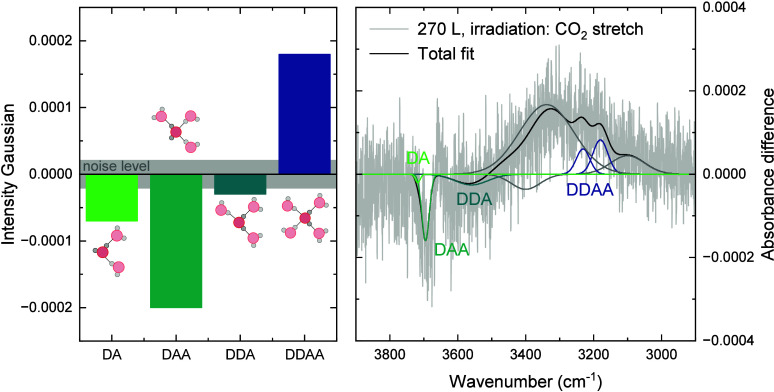
Absolute change in the intensity of the Gaussians parametrizing the different hydrogen-bonding surroundings (sketches, red circles: oxygen atoms, gray circles: hydrogen atoms, dashed lines: hydrogen bonds) for the irradiation of the CO_2_ stretch on 360 L ice. The gray band labeled noise level in the left panel indicates the average height of the Gaussians when fitting a noise region. The right panel shows the fit of the experimental data with the 8 Gaussians. The DDAA environment is described by two Gaussians.

### 3.3 Irradiation-Induced Changes in the CO_2_ Asymmetric Stretch

Interestingly, the experimental difference spectra show a change in the restructuring profile observed in the CO_2_ asymmetric stretch for ices thicker than 53 L, where the restructuring becomes a blueshift instead of a redshift. This is indicated with blue and red arrows in Figure 4b). These two different restructuring shapes were previously observed for pure CO_2_,^33^ but there they were attributed to irradiation at different resonant frequencies, whereas here they seem to be connected to the thickness of the ice.

Shapes analogous to the blueshift and redshift in the irradiation difference spectra are also observed in the changes induced by global heating of the substrate when performing TPD experiments, as can be seen in Figure 6 for a 27 and 360 L ice. The difference spectra in Figure 6a) and b) are obtained from separate control experiments on unirradiated ices by subtracting the spectrum of an ice at elevated temperatures from a cold ice spectrum at 10 K. For both ice thicknesses, a clear change in the shape of the heating-induced restructuring can be observed for a temperature increase of more than 40 K, where the negative intensity and positive intensities switch positions. Since we start our TPD experiments at 10 K, the 40 K temperature increase matches the 50 K mark at which crystalline CO_2_ features were observed for pure CO_2_ ^37^ and pure CO_2_ ice enters a polycrystalline regime. We will denote the profile observed upon heating with less than 40 K as low-temperature profiles and heating with more than 40 K as high-temperature profiles.

**Figure 6:**
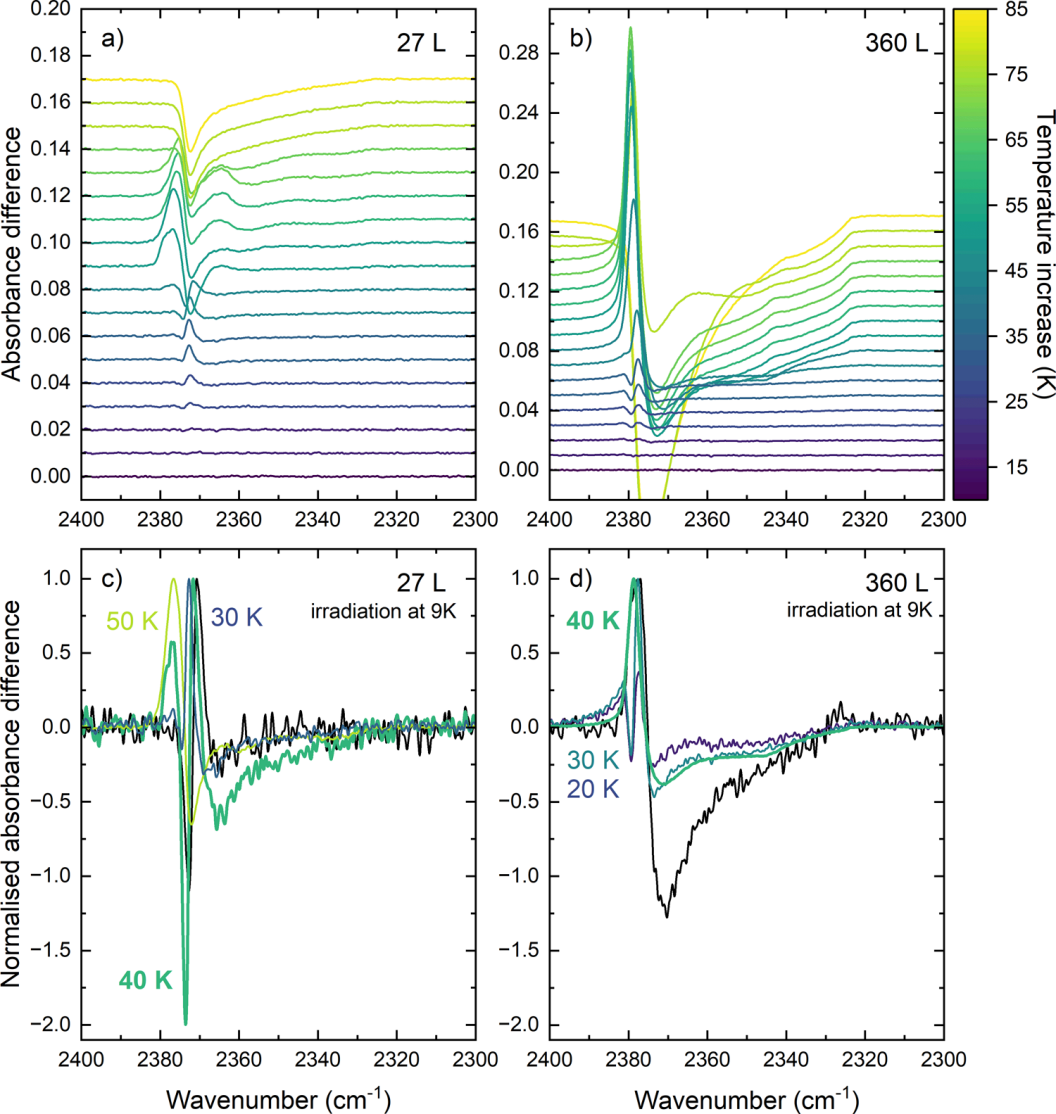
Comparison of TPD experiments with the red-shift and blue-shift restructuring profile. Difference spectra between a spectrum at 10 K and at elevated temperature are shown for a) the thinnest ice of 27 L and b) the thickest ice of 360 L. The color bar indicates the temperature increase from 10 K. The normalized difference spectra after irradiation at the CO_2_ asymmetric stretch are shown in black for c) the 27 L ice together with relevant normalized difference spectra from panel a) and for d) the 360 L ice with relevant normalized difference spectra from panel b).

The TPD difference spectra show clear differences between the heat-induced changes in a 27 L compared to a 360 L ice. In particular, the low-temperature profiles of both ices are significantly different. Where the 27 L ice presents a mainly ‘down–up–down’ profile (from left to right), the 360 L ice presents an ‘up–down–up–down’ profile. The differences in the high-temperature profiles of both thicknesses are more pronounced, with a clear extra upward peak at 2365 cm^–1^ in the profile of the 27 L ice, which is almost absent for the 360 L ice and only appears for a temperature increase of 75 K just before desorption.

These differences between a thick and a thin ice upon heating of the substrate are likely the result of the poor heat conductivity in a thick ice. This is confirmed by the slightly higher onset temperature of the desorption for the 360 L ice compared to the 27 L ice when studying the MID traces recorded during the TPD. Despite the delayed desorption of the 360 L ice, this thick ice seems to convert to a high-temperature ‘down–up’ profile at lower temperatures compared to the 27 L ice. This can be related to the higher bulk-to-surface ratio for the thicker ice, facilitating the process of crystallization in the bulk of the ice.

Figure 6c) and d) compare the observed redshift and blueshift profiles for the irradiations with the characteristic high-temperature and low-temperature profiles of the TPD experiments in Figure 6a) and b). All difference spectra are normalized with the intensity of their positive component for easier comparison. The observed redshift of the 27 L ice can be linked to a global temperature increase between 20 and 40 K. The slight shift in the positive peak position of 2 cm^–1^ is related to the TPD being recorded from another ice deposition. For the 360 L ice, the shape seems to correspond best to a high-temperature profile, indicating a temperature increase above 50 K, where, for pure CO_2_, the ice structure becomes polycrystalline. The match is not perfect, and for both ices, the irradiation of the stretch shows a larger negative contribution than that of the TPD difference spectra. This suggests that the on-resonance irradiation does not only induce global heating, and the TPD difference spectra cannot fully fit the irradiation-induced changes. This is also previously observed for the irradiation of pure CO_2_.^33^

### 3.4 Simulated Vibrational Excitation

The experimental results show an interaction between irradiation of the CO_2_ modes and changes in the OH stretch of H_2_O. We will investigate the nature of this interaction in more detail with Molecular Dynamics simulations on a simulation box with periodic boundary conditions, in line with the bulk nature of our experimental ice samples. To study dissipation pathways both within and between chemical species, we model a cluster of nonexcited molecules in an excited surrounding. Any changes occurring in this cluster after excitation of the surroundings can then be attributed to the dissipation of the vibrational energy into the vibrational modes of the molecules in the cluster. This is a different approach compared to the experiment, where only a small number of molecules is excited and dissipates its energy to a larger body of molecules. Yet, since our focus is to track the dissipation of energy, detection is clearer when many excited molecules dissipate to a small cluster of unexcited molecules, resulting in a larger energy increase in the small cluster.

Using the classical Molecular Dynamics simulations we neglect nuclear effects and, apart from zero-point energy effects, this means that the modeled dissipation is not quantized and also fractions of photon energy can transfer between different modes. In reality, this will not occur and the quantized nature of dissipation will keep the energy more localized. However, we do not expect the possible dissipation channels to be different. Our classical results already show strong selection rules based on spectral overlap, which is also highlighted by simulations on energy dissipation in pure ASW.^21,25^ Methods that can treat these nuclear quantum effects are computationally too demanding to treat the large number of molecules and the long time scale needed to relate to the laboratory experiments.

There are many ways to track the vibrational energy in molecular dynamics simulations, but to relate this to the experimental data discussed in this paper, we choose to display the kinetic energy in the vibrational modes through the mass-weighted vibrational spectrum. This spectrum shows all vibrational modes of the system and the intensity is proportional to the kinetic energy in that mode. This means that it cannot be directly compared with the experimental infrared spectra, since the intensities of the experimental RAIR spectra are related to the change in the dipole moment and thus exclude symmetric stretches and favor H_2_O over CO_2_ modes.

Figure 7 shows the summed vibrational spectrum for a group of 50 initially unexcited molecules calculated for three different situations; (i) no excitation during the simulation in red, (ii) excitation of all other molecules around the 50 molecules with the CO_2_ bending vibration (661.38 cm^–1^) in green and (iii) no excitation, but the simulated ice is kept at the elevated temperature of roughly 51 K reached during (ii) in blue. Situation (iii) is studied to help distinguish the on-resonance effects of the excitation with the electric field from a general heating of the simulated ice. The different panels show the vibrational modes of H_2_O and CO_2_, labeled above the panels. The frequency of the excitation is indicated by the dashed vertical line. The vibrational modes are slightly different for each H_2_O in the cluster because of their different local environment, which results in the ‘peaky’ structure of these modes. The CO_2_ vibrational modes are much more homogeneous, resulting in clear, well-defined features.

**Figure 7:**
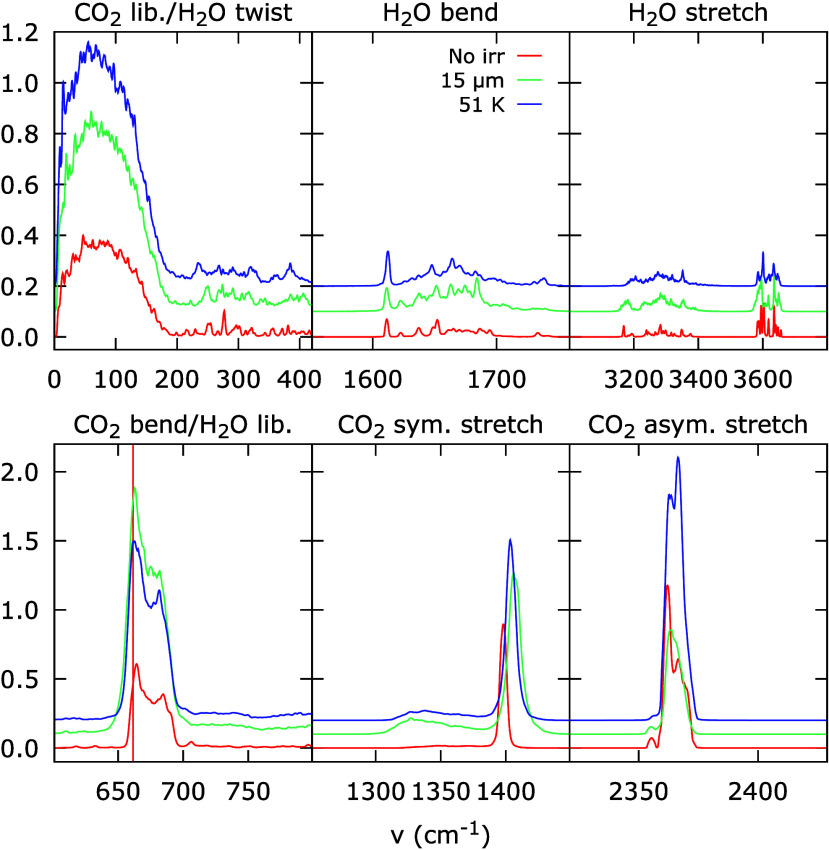
Summed vibrational density of states (VDOS) for a cluster of 50 molecules in a simulated 1:4 H_2_O:CO_2_ mixture. The different panels show the regions of the VDOS containing the relevant vibrational modes, labeled on top of the panels, with the H_2_O modes in the top row and the CO_2_ modes in the bottom row. The VDOS is calculated for three different situations: red (‘No irr’), no excitation is applied to the cluster or the rest of the simulation box; green (‘15 μm’), an excitation of the CO_2_ bending vibration (indicated with a dashed vertical line) is applied to the entire simulation box except the cluster; and blue (‘51 K’), the cluster is only heated to the temperature reached after the excitation of the surrounding.

Thermally heating the cluster of molecules as in situation (iii) naturally leads to an increased intensity in all vibrational modes, since the intensities of the peaks are directly proportional to the kinetic energy, as they are determined from the velocity autocorrelation. The green curves in Figure 7 for the excited system show that the increase in vibrational energy in the bending vibration is much stronger than in the other modes. Moreover, the increase is stronger than for heating of the system. This suggests that excitation is dissipated on-resonance from excited to nonexcited CO_2_ molecules. Furthermore, we observe that a bump appears next to the symmetric stretch of CO_2_ upon excitation of the surrounding molecules. This lower wavenumber peak at 1310 cm^–1^ corresponds to the double frequency overtone of the bending vibration that is excited in the simulation. This seems to interact with the symmetric stretching vibration, as both the intensity and position change after excitation. The effect is also observed when heating the system to 51 K, as indicated in blue, but not as strong as through the direct excitation of the CO_2_ bending mode. Because the symmetric stretch is infrared inactive, we are unable to observe this interaction directly in the experiments.

Related to this, we observed in our simulations that a slight degeneracy between the two CO_2_ bending modes within the same molecule can lead to an additional oscillation modulation of the difference frequency between the two degenerate modes. Since this is resonant with the CO_2_ libration frequency in the far-infrared, we suspect that this facilitates the dissipation to the intermolecular modes, resulting in a heating of the whole system. Indeed, for all vibrational modes, a small increase in intensity is observed with respect to the nonirradiated case. Even for the H_2_O libration mode, this increase can be observed in a slight offset in the baseline next to the CO_2_ bending vibration. When comparing the green trace for the vibrational excitation and the blue trace of the global heating, this increase in all vibrational modes of H_2_O is almost identical. This suggests that the H_2_O molecules are in some way thermally heated by the vibrationally excited CO_2_ molecules in the surroundings. As such, the simulations present an indirect on-resonance interaction between the CO_2_ bending mode and the H_2_O vibrational modes. Considering that the spectrum acquisition – lasting for 200 ps – starts 10 ps after the excitation, the simulations also reveal that the dissipation occurs on a relatively short time scale. Within these 200 ps the excitation has traveled from the excited environment to the cluster and dissipation to other modes has started.

We perform the same type of simulation for excitation of the CO_2_ asymmetric stretching vibration of all molecules except the cluster of 50 CO_2_ molecules. Figure 8 shows the resulting VDOS for a situation without excitation during the simulation in red and with excitation of all other molecules around the 50 molecules with the CO_2_ asymmetric stretching vibration (2361.8 cm^–1^), indicated by the dashed vertical line, with different delay times between excitation and calculation of the VDOS. The 100 ps delay time in green is already 10 times longer than that used for the simulations of the excitation of the bend in Figure 7. The different panels again show all vibrational modes. In this case, the intensity of the asymmetric stretch is divided by four to allow visibility of the other vibrational modes of CO_2_.

**Figure 8:**
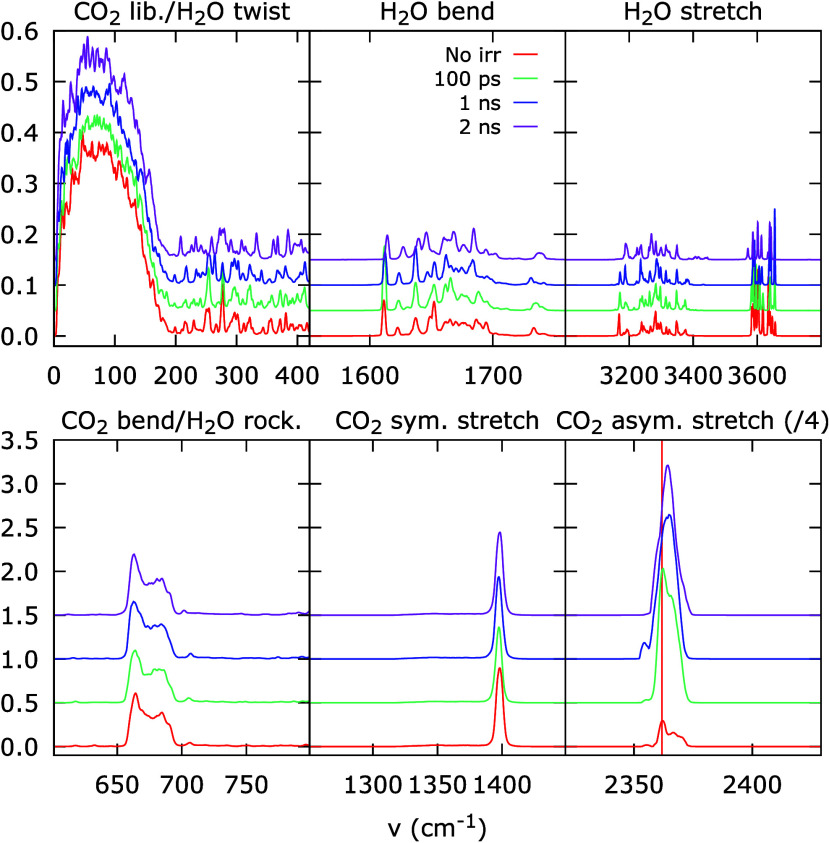
Summed and mass-weighted vibrational density of states (VDOS) for a cluster of 50 molecules in a simulated 1:4 H_2_O:CO_2_ mixture. The different panels show the regions of the VDOS containing the relevant vibrational modes, labeled on top of the panels, with the H_2_O modes in the top row and the CO_2_ modes in the bottom row. The red trace (‘No irr’) is calculated after a simulation where no excitation is applied to the cluster or the rest of the simulation box. The other three traces correspond to an excitation of the CO_2_ asymmetric stretching vibration (indicated with a dashed vertical line) applied to the entire simulation box except the cluster, but with different time delays between the excitation and the determination of the VDOS of 100 ps (green), 1 ns (blue), and 2 ns (pink).

A comparison of the red and green curves shows that no changes are observed with respect to the nonirradiated situation in any of the H_2_O or CO_2_ vibrational modes, except for the strongly excited asymmetric stretch. As such, energy is clearly dissipated on-resonance from the CO_2_ asymmetric stretch in the surroundings to the cluster of molecules. The VDOS spectrum at a delay time of 1 ns shows that the asymmetric stretch remains excited, but a slight broadening has occurred, hinting at some anharmonicity in the vibration. Eventually, this anharmonicity will lead to dissipation into other modes. Some slight shifts in the H_2_O twist can be observed, as well as a slight increase in intensity for the CO_2_ libration mode (3% increase of the area in the 0–500 cm^–1^ range). This trend is extended at a delay time of 2 ns with an increase of 12% in the area, but the process is extremely slow. This indicates that the asymmetric stretch is strongly isolated and is unable to dissipate its energy effectively to any other mode. However, the experiments measure the changes on a much longer time scale, where the simulations hint that this inefficient dissipation can have occurred.

## 4 Discussion

For the experiments, the strongest changes are observed for the irradiation of the CO_2_ asymmetric stretch, whereas in simulations excitation of the CO_2_ asymmetric stretch shows energy dissipation solely from one excited asymmetric stretch to another on short time scales. After two nanoseconds, the asymmetric stretch is still strongly excited in the simulations, whereas for the CO_2_ bending vibration the energy is easily dissipated further and thermal heating of the H_2_O vibrational modes is observed. Only after 2 ns a hint of anharmonicity in the asymmetric stretch appears to be coupled to the H_2_O twist and the CO_2_ libration, possibly allowing for the restructuring observed in the experiments on significantly longer time scales.

The pulsed nature of FEL-2 allows for about one nanosecond of relaxation time for the experimentally irradiated ice, which in the case of an irradiation of the CO_2_ bending mode would lead to a complete relaxation of the system and dissipation of energy to the intermolecular and H_2_O modes. However, in the case of an excitation of the asymmetric stretch, the simulations show that one nanosecond is not enough to relax the system between the FEL pulses. Instead, the system is still excited when the next infrared pulse hits, leading to a stacking of the excitation. Ladder climbing has been observed for high-intensity irradiation of the asymmetric stretching vibration of CO_2_ ^38^ and this could lead to a strong local heating of the ice structure that could result in the observed experimental changes in the OH stretch in addition to the very slow dissipation process also found in the simulations.

It should be noted that the absence of clear changes in the OH stretch after experimental irradiation of the CO_2_ bending vibration may have an experimental origin. When calculating the number of photons absorbed per molecule, as shown in the Supplementary, we find that for irradiation performed at 4.215 μm, the molecules receive a factor 10 more photons compared to irradiations at 14.88 μm. As a result, the experiment creates an artificial weakening of the changes observed when irradiating the bend. At the moment we cannot circumvent this artificial weakening, as experiments with higher fluences at the bend are currently not possible with the LISA setup and FEL-2.

Instead, in a quick control experiment on the 360 L ice in a separate beamshift, we attenuated the irradiation intensity of the FEL. In the current setup, this can only be done in rather coarse steps (3 dB, half the power) and this resulted in roughly a factorof two times more photons for the asymmetric stretch, compared to the bending mode as in Figure 4. The comparison in the Supplementary shows that for a reduced FEL intensity, the changes weaken, roughly linearly with the number of photons absorbed. Also, the changes in the OH stretch are weakened for this irradiation, suggesting that the irradiation of the bend could experimentally result in identical changes as for the stretch if the FEL could supply high enough power to match the ∼0.29 photons per molecule absorbed of the irradiation of the asymmetric stretch. It is however technically not possible for FEL-2 to provide >300 mJ in this wavelength range.

In addition, experiments that change the delay time between the FEL-2 pulses would be instrumental in testing the stacking of the vibrational energy upon excitation of the CO_2_ asymmetric stretch. We managed to perform some preliminary experiments using the 50 MHz mode of FEL-2, resulting in a time delay between micropulses of 20 ns instead of 1 ns. As shown in the Supplementary, this results in an almost equally strong change in the OH stretch for the largely equal irradiation strength, but the change in the CO_2_ asymmetric stretch is weakened. The remaining change in the asymmetric stretch resembles that of the irradiation of the CO_2_ bending vibration. This suggests that the stacking of irradiation energy in the CO_2_ asymmetric stretch due to insufficient relaxation between micropulses does not play a major role in the observed changes in the OH stretch. Instead, the slow increase in anharmonicity after excitation appears to be the dominant pathway toward excitation of H_2_O, as this would be independent of the time delay between the micropulses. Both additional experiments reported here briefly are preliminary, and more detailed studies on both the irradiation intensity dependence and the relaxation time between micropulses are required to fully understand the difference observed in the simulations and experiments.

## 5 Astrophysical Implications

Our results show that energy dissipation can be rapid provided that there is spectral overlap between the excited vibration and the other molecules in the ice, mainly H_2_O. We can highlight the relevance of this observation if we return to the example that we posed in the Introduction, namely, the HOCO complex formed in the CO + OH reaction and efficiently stabilized through the dissipation of energy. Upon formation, the HOCO molecule is likely excited in one or more of the modes connected to the formed HO–CO bond at 1279, 1084, 605, and 538 cm^–1^.^39,40^ Those at 1084, 605, and 538 overlap with vibrational modes of H_2_O and, based on our current studies, rapid energy dissipation is expected. The consequence of this is that HOCO does not directly proceed to the formation CO_2_ + H as is the case in the gas phase, but a subsequent H atom is required to react to CO_2_ + H_2_, HCOOH, and H_2_O + CO. Since there is no spectral overlap between a CO ice and the HOCO modes, we expect the stabilization of HOCO in a CO-rich environment to be much less efficient. This is in agreement with earlier studies.^18,41^ Whether HOCO is stabilized or not will affect the final CO_2_:HCOOH:H_2_O abundance ratios. These consequences are not limited to the CO + OH reactions, and radical–radical reactions will typically also result in highly excited new molecules and the dissipation of this energy is also crucial in the survival of large newly formed molecules versus dissociation to smaller compounds.

The dissipation of energy from the CO_2_ vibrational modes to H_2_O, as observed in this paper, results in increased ordering of the hydrogen-bonding network in the ice, the result of which looks spectroscopically like annealing of the ice. With this work, we have thus experimentally found a nonthermal pathway that can lead to annealing of the H_2_O ice on the interstellar dust grain. We consider this pathway nonthermal as it is an on-resonance process and initially depends on the excitation of one specific vibrational mode, instead of the excitation of all vibrational modes as for thermal heating. Previous work on pure pASW ices has also shown nonthermal annealing pathways when vibrationally exciting the OH stretch.^21,25^ In that sense, not only the thermal history of the ice should be considered to explain the characteristics of annealing in the H_2_O spectral features, but also the possibility of exposure to mid-infrared irradiation, either directly on-resonance with the H_2_O vibrational modes, or those of CO_2_.

The experiments also show that the dissipation efficiency from CO_2_ to H_2_O is subject to a slight thickness dependence. Only the thicker ices show clear restructuring in H_2_O. In interstellar space, one expects the thickest ices in the coldest regions, such as dense cores in molecular clouds and the midplanes of protoplanetary disks. These regions are ideal for thick ice, but are strongly shielded from the interstellar radiation field. However, unlike UV radiation, infrared radiation is hardly shielded and still penetrates deep into these dense cores. Therefore, in these regions with thick ice, we can expect the infrared-induced processes we observed in our experiments and simulations to dominate over any UV-induced processes. In addition, excitation of the CO_2_ vibrational modes leads to an on-resonance and indirect heating of the ice that in turn could lead to more diffusion, facilitating chemical reactions in the ice.

Considering the time scale of the infrared irradiation, our experiments reveal an interaction between infrared excitation of the CO_2_ asymmetric stretch, while our simulations only show such a connection for excitation of the bending vibration when considering short time scales. Consequently, on short time scales of a few hundred picoseconds, an excitation of the CO_2_ bending vibration is most efficient in thermally altering the H_2_O structure. On long time scales with repeated excitations over the course of tens of seconds, the asymmetric stretching vibration of CO_2_ also offers a way to interact with H_2_O, possibly through ladder climbing, but mainly through the very slow anharmonicity induced transfer of energy from the asymmetric stretch to the CO_2_ lattice modes and H_2_O twist modes. In interstellar space, the infrared photon flux is easily orders of magnitude lower than that of FEL-2. This corresponds to a longer time interval between incoming photons that could possibly hamper the experimentally observed interaction between the CO_2_ asymmetric stretch and the H_2_O vibrational modes, and the faster indirect pathway through the CO_2_ bending mode may be dominant. Yet, preliminary experiments suggest that the time interval between incoming photons is not the main driver of the energy dissipation from the CO_2_ asymmetric stretch to H_2_O.

## 6 Conclusions

In the research presented here, we have investigated the effect of infrared irradiation on-resonance with the CO_2_ asymmetric stretching and bending vibration on a mixed H_2_O:CO_2_ 1:4 ice both experimentally and through Molecular Dynamics simulations. The combination of results from both the experiment and simulations allows us to match the macroscopic processes with molecular processes and access the structural changes in the ices at different time scales. Our main findings are as follows:

1. Experimentally, changes are only observed clearly when irradiating the CO_2_ asymmetric stretching vibration, which is likely a result of the 10 times more photons per molecule absorbed in this vibrational mode compared to the CO_2_ bend;
2. The changes are characterized by a red-shift in the CO_2_ asymmetric stretch for the two thinnest ices of 27 and 53 L and a blue-shift for the thickest ices that seems to be related to low temperature (<40 K) induced changes for the thin ices and a high temperature (>40 K) induced change for the thick ices, where the high temperature induced changes are likely connected to polycrystalline CO_2_;
3. Additionally, the irradiation of the CO_2_ asymmetric stretching vibration results in desorption of CO_2_, but also in a clear increase in intensity in the OH stretch of water, signaling segregation.
4. In the simulations, energy dissipation on-resonance with the same vibrational mode of a neighboring nonexcited molecule is observed for both the excitation of the CO_2_ bending vibration and asymmetric stretching vibration;
5. The asymmetric stretching of CO_2_ is an isolated mode on short time scales – it does not dissipate to or couple with other vibrations in the mixed system – and this vibrational mode stays excited for more than 2 ns after excitation;
6. On longer time scales, the prolonged excitation of the asymmetric stretch results in an increased anharmonicity in the mode, resulting in a slow dissipation of energy into the CO_2_ libration and H_2_O twist mode, possibly initiating the structural changes observed experimentally in the hydrogen-bonding network.
7. From an excitation of the CO_2_ bending vibration, the energy is readily distributed and coupled with the intermolecular interactions that lead to thermal heating of the H_2_O vibrational modes on short time scales.

Despite the apparent differences between the characteristics of the simulations and experiments reported here, the combination of both methods has revealed an interaction between the CO_2_ and H_2_O vibrational modes that, depending on the time scale, occurs via an increased vibrational anharmonicity in the CO_2_ asymmetric stretch or the coupling of the CO_2_ bending vibration to the intermolecular modes that induces a local heating. Both processes can result in the restructuring of the H_2_O molecules in the mixed system. Infrared irradiation can thus be considered a nonthermal pathway for the annealing of H_2_O ice in space, even when the infrared field is not resonant with the H_2_O vibrational modes, but with those of CO_2_. Ideally, time-resolved experiments should be performed to increase the overlap between experiment and simulation, and such experiments are planned to be developed for the LISA setup in the future.

## Supplementary Material



## Notes

Additional experimental information including the determination of the deposition time corresponding to a single monolayer of CO_2_ (S1, Figure S1), multiple ion detection (MID) traces recorded during the irradiation experiments (S2, Figures S2, S3, and S4), the estimation of the number of photons absorbed per molecule for irradiation at the CO_2_ asymmetric stretching and CO_2_ bending vibration (S3), and the results of preliminary experiments changing the time between the FEL macropulses and the irradiation intensity of the FEL (S4, Figure S5) (PDF)
