# Supplementary material for: Infrared Irradiation of H2O:CO2 Ice: A Combined Experimental and Computational Study of the Dissipation of CO2 Vibrational Excitations
Source: ACS Earth Space Chem. 2025 May 23;9(6):1580–92. doi: 10.1021/acsearthspacechem.5c00030 (PMC12183729; doi:10.1021/acsearthspacechem.5c00030)
Supplement: Supplementary file 1 [file sp5c00030_si_001.pdf]

Supporting Information
Infrared Irradiation of H_2O:CO_2 Ice –A Combined Experimental and Computational Study of the Dissipation of CO_2 Vibrational Excitations–

Johanna G. M. Schrauwen,[†] Tobias M. Dijkhuis,[‡,¶,§] Sergio Ioppolo,[∥] Daria R. Galimberti,[⊥] Britta Redlich,[†] and Herma M. Cuppen[*,⊥]

†*HFML-FELIX Laboratory, IMM, Radboud University, Toernooiveld 7, 6525 ED Nijmegen, The Netherlands*

‡*Institute of Molecules and Materials (IMM), Radboud University, 6525 ED Nijmegen, The Netherlands*

¶*Leiden Institute of Chemistry, Gorlaeus Laboratories, Leiden University, 2300 RA Leiden, The Netherlands*

§*Leiden Observatory, Leiden University, 2300 RA Leiden, The Netherlands*

∥*Centre for Interstellar Catalysis (InterCat), Department of Physics and Astronomy, University of Aarhus, Aarhus DK-8000, Denmark*

⊥*Institute of Molecules and Materials (IMM), Radboud University*

E-mail: h.cuppen@science.ru.nl

S1 Deposition time of a CO_2 monolayer

Upon the change in desorption from a sub-monolayer to a multilayer ice, a characteristic change can be observed in the temperature programmed desorption (TPD) profile. As shown in Figure S1, a number of depositions with varying deposition time are performed and the TPD spectra are recorded with a temperature ramp of 5 K/min. To speed up the measurements, the first part of the TPD from 10 to 40 K is performed at the maximum capacity of the heater without a preset ramp. The depositions of roughly 2.7 L show a double-peak feature, which we interpret as an indication of the transition from monolayer to multilayer desorption.

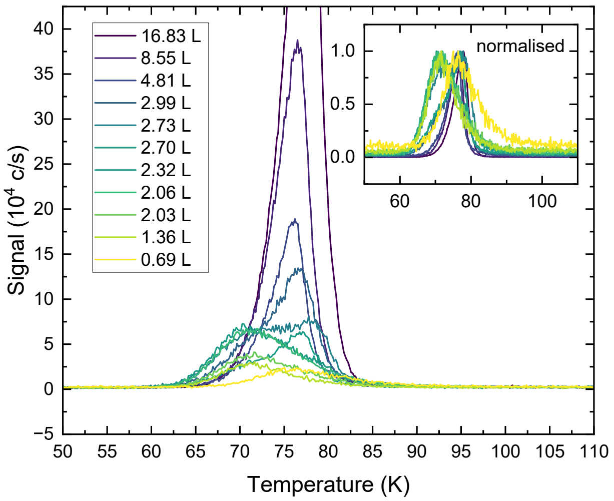

Figure S1: Temperature programmed desorption traces of CO_2 at 5 K/min for depositions of 0.7 to 16 L (yellow to blue). Exact deposition times are shown in the figure legend. The inset shows the normalised curves.

S2 Desorption of CO_2

During the irradiation experiments, the gas phase is monitored by the mass spectrometer in multiple ion detection (MID) mode. Both m/z 18 (H_2O) and m/z 44 (CO_2) are recorded with the dwell time and settle time of the mass spectrometer set to 5 ms. Then both masses are recorded almost 50 times per second, while the FEL-2 macropulses irradiate the substrate at 10 Hz, resulting in a total of 5 data points for both masses per macropulse. For most irradiations, no significant signal above the noise was observed in the MID trace. Figures S2, S3, and S4 only show the MID traces that recorded some kind of desorption. Figure S2 for the irradiation at the CO_2 asymmetric stretch on the thickest ice of 360 L shows some spikes of decaying intensity. The smallest spacing between the spikes is 0.1 s, corresponding to the 10 Hz repetition rate of the macropulse. However, as can be seen in the inset in Figure S2, a desorption spike cannot be observed for every macropulse. This can have a physical origin, but could also be a mismatch between the recording frequency and the pulse frequency, since the mass spectrometer is not synchronised with the FEL-2 pulses. The other two figures only show one desorption spike at the start of the irradiation. For all irradiations, no desorption was observed for m/z 18.

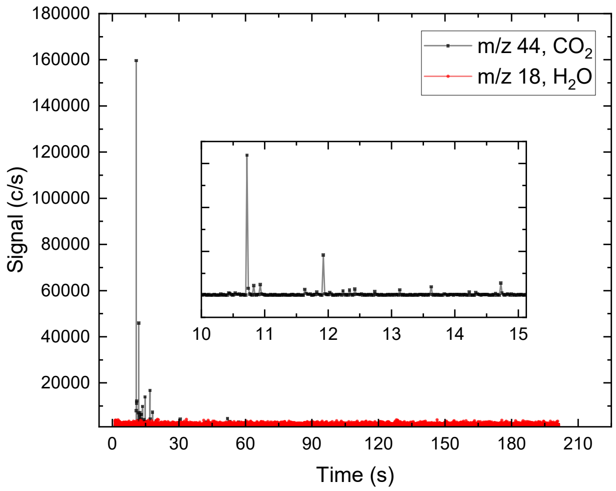

Figure S2: MID trace for m/z 18 (red) and m/z 44 (black) recorded during irradiation at CO_2-stretch on the 360 L ice. The first spike is at the start of the irradiation. The inset shows a zoom-in on the first few spikes recorded.

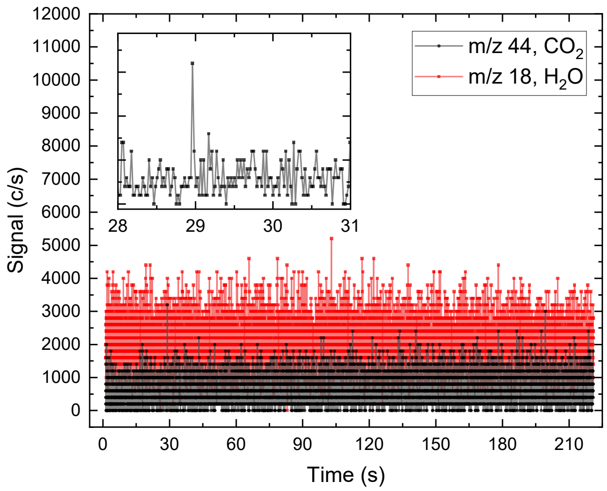

Figure S3: MID trace for m/z 18 (red) and m/z 44 (black) recorded during irradiation at CO_2-bend on the 107 L ice. The inset shows a zoom-in of the spike at the start of the irradiation.

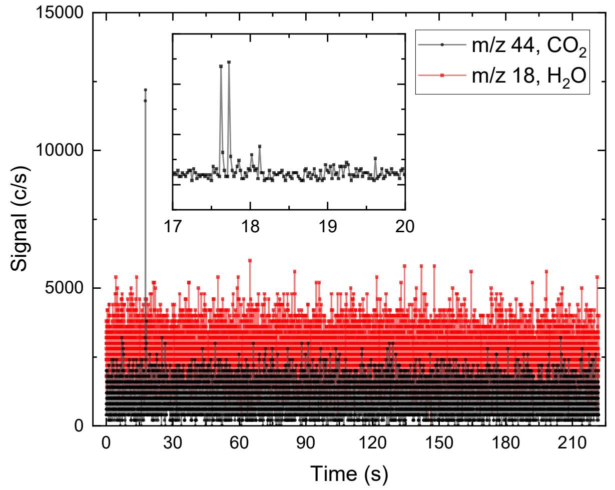

Figure S4: MID trace for m/z 18 (red) and m/z 44 (black) recorded during irradiation at CO_2-bend on the 360 L ice. The inset shows a zoom-in on the few spikes at the start of the irradiation.

S3 Number of photons per molecule absorbed

Here, we calculate the number of photons absorbed by a monolayer of H_2O:CO_2 1:4 ice analogue upon irradiation of the stretching vibration and the bending vibration of CO_2. Using the irradiation characteristics of 67 mJ at 4.215 μm and 90 mJ at 14.88 μm, the irradiation supplies $1.42 \cdot 10^{18}$ and $6.4 \cdot 10^{18}$ photons per macropulse of 6 μs for irradiation at stretch and bend, respectively. Considering the 1 ns time between micropulses in the macropulse, we can expect the ice to have relaxed after a single micropulse for the excitation of the bending mode, whereas this is likely not the case for the stretching mode. In the latter case, we can have a stacking of the vibrational excitation as discussed in the main text. Still, to obtain comparable values for the excitation of both vibrational modes, we only consider the micropulse in the following estimation.

With 6000 micropulses in a macropulse for both irradiations, this results in $2.37 \cdot 10^{14}$ and $1.12 \cdot 10^{14}$ photons per micropulse for irradiation at the stretch and bend, respectively. The spot size of FEL-2 at 4 μm is 0.22 mm^2 and at 15 μm is 1.71 mm^2, which, using the general molecular surface density of 10^{15} cm^2, results in a total of 104 and 66 photons per molecule per micropulse for irradiation at stretch and bend. Not all of these photons are absorbed by the molecules, and to estimate the absorbed fraction we use $\alpha(\nu) = 4\pi k\nu$ with k the extinction coefficient. With extinction coefficients of 2.8 at 2350 cm^{-1} and 2.2 at 662 cm^{-1},[1] this results in absorbed fractions in the top layer of 3.5 Å of $1 - \exp(-8.3 \cdot 10^4 \cdot 3.5 \cdot 10^{-8}) = 2.9 \cdot 10^{-3}$ and $1 - \exp(-1.8 \cdot 10^4 \cdot 3.5 \cdot 10^{-8}) = 6.3 \cdot 10^{-3}$ for irradiation at the stretch and bend, respectively. Then, the number of photons absorbed per molecule in the top layer is 0.3 for the CO_2 asymmetric stretch and 0.04 for the CO_2-bending vibration.

To compare these absorbed photons to the electric field amplitude we used for the simulations ($4.0 \cdot 10^8$ V/m for the stretching mode and $2.0 \cdot 10^8$ V/m for the bending mode) we can calculate the intensity of a single micropulse in W m^{-2} and convert to an electric field strength using the average Poynting vector. For the CO_2 stretching vibration, the intensity I is

$$I = \frac{E_{\text{macro}}}{N_{\text{micro}} A t_{\text{micro}}} = \frac{0.067}{6000 \cdot 0.2 \cdot 10^{-6} \cdot 4 \cdot 10^{-12}}$$

which is $1.3 \cdot 10^{13}$ W m^{-2}. Using the average Poynting vector the electric field amplitude is

$$E_{0,z} = \sqrt{\frac{2I}{\epsilon_0 c}}$$

which is $9.8 \cdot 10^7$ V/m for the stretching vibration. For the CO_2 bending vibration we get

$$E_{0,z} = \sqrt{2 \cdot 2.2 \cdot 10^{12}/(\epsilon_0 c)}$$

amounting to $4.1 \cdot 10^7$ V/m. For the irradiations of CO_2 we chose stronger fields in the simulations to enhance the traceability of the energy dissipation.

S4 Preliminary experiments: 50 MHz and low-intensity irradiations of the 360 L ice

The results in Figure S5 are preliminary and study the effect of reduced irradiation intensity (green curve, 0.08 absorbed photons per molecule) and a longer relaxation time between micropulse (blue-green curve, 0.20 absorbed photons per molecule, 50 MHz). The latter is performed with a FEL micropulse frequency of 50 MHz, instead of 1 GHz, but with a 20 times longer irradiation time to maintain the irradiation intensity of the original experiment on the 360 L ice. In principle, this irradiation supplies an identical number of photons per molecule, but the micropulses are spaced 20 ns apart, instead of 1 ns. Figure S5 shows that we do not observe a significant difference in the changes in the OH stretch of H_2O of the 50 MHz irradiation compared to the 1 GHz irradiation.

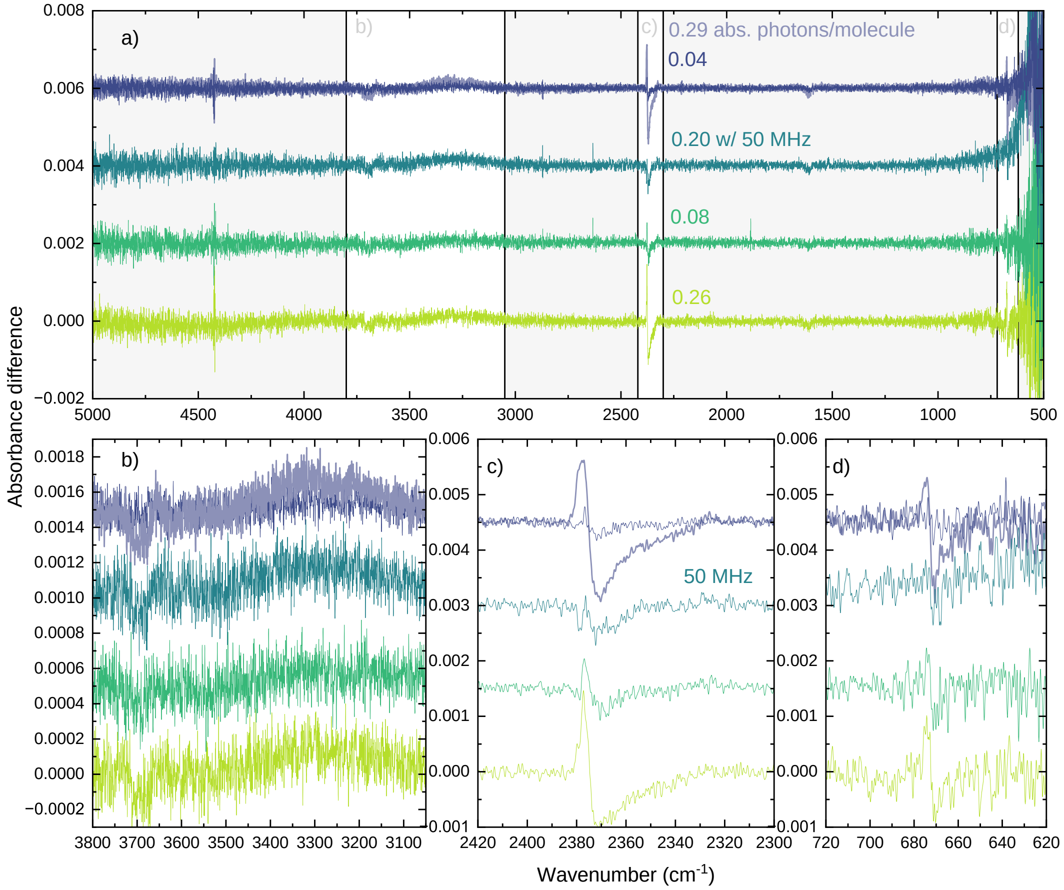

Figure S5: Infrared difference spectra for two additional FEL settings for irradiation of the CO_2 asymmetric stretch on the 360 L ice. Panel a) shows the full difference spectra and b), c) and d) zoom in on the changes in the relevant vibrational modes, being the OH-stretch of H_2O, the CO_2-stretch and the CO_2-bend, respectively. The top two traces are overlaid and identical to the irradiations of Figure 4 in the manuscript. They are included for comparison with an irradiation of identical conditions (light-green trace, bottom, 0.26 absorbed photons per molecule), and irradiation with reduced irradiation intensity (green trace, 0.08 absorbed photons per molecule) and an irradiation with an increased relaxation time between the micropulses (blue-green trace, 0.20 absorbed photons per molecule, 50 MHz). The difference spectra are offset vertically for clarity.

References

(1) Ehrenfreund, P.; Boogert, A. C. A.; Gerakines, P. A.; Tielens, A. G. G. M.; van Dishoeck, E. F. Infrared spectroscopy of interstellar apolar ice analogs. *Astronomy and Astrophysics* **1997**, *328*, 649–669.
